# Macrophages and Monocytes: “Trojan Horses” in COVID-19

**DOI:** 10.3390/v13112178

**Published:** 2021-10-28

**Authors:** Elena Percivalle, Josè Camilla Sammartino, Irene Cassaniti, Eloisa Arbustini, Mario Urtis, Alexandra Smirnova, Monica Concardi, Cristina Belgiovine, Alessandro Ferrari, Daniele Lilleri, Antonio Piralla, Fausto Baldanti

**Affiliations:** 1 Molecular Virology Unit, Microbiology and Virology Department, Fondazione IRCCS Policlinico San Matteo, 27100 Pavia, Italy; e.percivalle@smatteo.pv.it (E.P.); jose.sammartino@iusspavia.it (J.C.S.); alessandro.ferrari04@universitadipavia.it (A.F.); d.lilleri@smatteo.pv.it (D.L.); a.piralla@smatteo.pv.it (A.P.); fausto.baldanti@unipv.it (F.B.); 2 Transplant Research Area and Centre for Inherited Cardiovascular Diseases, Department of Medical Sciences and Infectious Diseases, Fondazione IRCCS Policlinico San Matteo, 27100 Pavia, Italy; e.arbustini@smatteo.pv.it (E.A.); m.urtis@smatteo.pv.it (M.U.); a.smirnova@smatteo.pv.it (A.S.); m.concardi@smatteo.pv.it (M.C.); 3 Humanitas Clinical and Research Center—IRCCS, 20089 Milan, Italy; Cristina.Belgiovine@humanitasresearch.it; 4 Department of Clinical, Surgical, Diagnostics and Pediatric Sciences, University of Pavia, 27100 Pavia, Italy

**Keywords:** SARS-CoV-2, Trojan horse, VERO E6 cells

## Abstract

We aimed to explore whether variants of SARS-CoV-2 (Chinese-derived strain (D614, lineage A), Italian strain PV10734 (D614G, lineage B.1.1) and Alpha strain (lineage B.1.1.7)) were able to infect monocytes (MN) and monocyte-derived macrophages (MDM) and whether these infected cells may, in turn, be vectors of infection. For this purpose, we designed an in vitro study following the evolution of MN and MDM infection at different time points in order to confirm whether these cells were permissive for SARS-CoV-2 replication. Finally, we investigated whether, regardless of viral replication, the persistent virus can be transferred to non-infected cells permissive for viral replication. Thus, we co-cultured the infected MN/MDM with permissive VERO E6 cells verifying the viral transmission. This is a further in vitro demonstration of the important role of MN and MDM in the dissemination of SARS-CoV-2 and evolution of the COVID-19 disease.

## 1. Introduction

Beta-coronaviruses are associated with human diseases, and, in the last two decades, the emergence of Middle East Respiratory Syndrome (MERS-CoV) and Severe Acute Respiratory Syndrome (SARS-CoV-1) viruses, with a zoonotic origin, were connected to two outbreaks of severe respiratory diseases in 2012 and 2003, respectively [1]. In late 2019, a novel coronavirus disease (COVID-19) caused by the new beta-coronavirus Severe Acute Respiratory Syndrome 2 virus (SARS-CoV-2) was reported in Wuhan (Hubei province, China). COVID-19 was declared as a pandemic by the World Health Organization on 11 March 2020 [2]. Despite the paramount scientific effort in the dissection of the pathogenetic mechanisms of COVID-19, virologic and immunologic factors triggering severe disease in SARS-CoV-2-infected subjects are not completely defined. In addition, while it is now evident that COVID-19 is a multi-organ disease [3], the virus dissemination mechanisms are not fully elucidated.

A common denominator of all infected organs in COVD-19 disease is the expression of angiotensin-converting enzyme 2 (ACE2), which was identified as the binding receptor for the spike viral glycoprotein [4] that allowed viral internalization and replication in the host cells. However, little attention has been given to ACE-2 expression in the immune system; indeed also, monocytes and macrophages [5] express this receptor, making them potential targets of the infection. Macrophages play an important role in host defense and are present within all tissues of the body, including the respiratory system. They are potent producers of cytokines that are crucial components of innate immunity and potential mediators of immunopathology [6]. So far, infected CD68+CD169+ macrophages were found in human thrombi recovered in vivo for clinical reasons (unpublished data) and in several infected organs [7], suggesting that these cells could contribute to the viral spread. Spleen and lymph nodes’ ACE2+ macrophages could be infected by SARS-CoV-2 [8], suggesting that they could be responsible for viral spread. While the infection of monocytes is abortive in SARS-CoV, MERS-CoV can replicate in monocytes, macrophages and dendritic cells [9,10]. Although SARS-CoV-2 viral particles were detected in monocytes and macrophages [11], the evidence of productive infection remains to be demonstrated.

In the present study, we investigated whether monocyte-derived macrophages (MDM) and monocytes (MN) were fully permissive for SARS-CoV-2 replication or, alternatively, were passive carriers of the virus and viral material disseminating the infection to multiple body sites, acting as a “Trojan horse”. In order to verify this hypothesis, we used an in vitro model previously developed for the generation of human cytomegalovirus (HCMV) pp65-positive polymorphonuclear leukocytes (PMNLs) [12]. In summary, through co-cultivation of MN and MDM with SARS-CoV-2-infected VERO E6 cells, we showed that the virus was transferred from infected VERO E6 to MN and MDM, even if abortive viral replication occurred in these cells. Finally, we proved that infected MN and MDM were able to a transmit the virus actively to uninfected VERO E6. Thus, we might conclude that abortively infected MN and MDM, as they occur for abortively infected PMNLs in HCMV disease, could play a crucial role for SARS-CoV-2 in the in vivo dissemination to multiple body sites.

## 2. Materials and Methods

### 2.1. SARS-CoV-2 Variants

SARS-CoV-2 strains, including the wild type Chinese-derived strain (D614, lineage A), Italian strain PV10734 (D614G, lineage B.1.1) and Alpha variant (lineage B.1.1.7), were isolated at 33 °C from infected patients’ nasal swabs in the permissive VERO E6 (VERO C1008 (Vero 76, clone E6, Vero E6; ATCC1CRL-1586TM) cell line [13]. Virus variants were titrated to prepare the cell-free virus for MN and MDM infection. Complete genome sequencing [14] was performed in order to confirm the presence of variant-defining mutations, and sequences were submitted to GISAID under the following reference numbers: EPI_ISL_568579; EPI_ISL_1403609-11.

### 2.2. Cell Culture

Peripheral blood mononuclear cells (PBMC) were isolated using Ficoll-Paque Plus (GE Healthcare, Chicago, IL, USA) from five discharged buffy-coat units of healthy blood donors, given by the “Blood Bank” Processing and validation center DMTE/SIMT Fondazione IRCCS Policlinico San Matteo, according to the Italian law decree of Ministry of Health, 2 November 2015 “Provisions relating to the quality and safety requirements of blood and blood components”. PBMC were plated at a concentration of 5 × 10^6^ into 24-well microplates (COSTAR, Corning Incorporated, Corning, NY, USA) for 3 h at 37 °C, 5% CO_2_ to let the cells adhere. Non-adherent cells were removed, and MDM were differentiated by stimulation with the Granulocyte-Macrophage Colony-Stimulating Factor (GM-CSF) at 800 U/mL for 14 days. In parallel, MDM were differentiated onto round glass coverslips placed in 24-well plates with a round glass coverslip for cell staining after virus infection. For MN experiments, after 3 h of adhesion, non-adherent cells were removed, and adherent cells were used for the experiments.

### 2.3. Flow Cytometry

The characterization of differentiated MDM was performed via flow cytometry with the following mAbs: CD11b BV 786 (Biolegend, San Diego, CA, USA; clone IRCF44), CD14 APC (BD Biosciences, Franklin Lakes, NJ, USA; clone M5E2), CD206 FITC (BD Biosciences, clone 19.2), HLA-DR BV711 APC (BD Biosciences, clone G46-6), CD16 PE (BD Biosciences, clone 3G8), CD4 BV 421 (BD Biosciences, clone RPA-T4), CD8FITC (BD Biosciences, clone RPA-T8), CD19 APC (BD Biosciences, cloneHIB19) and CD69PECy7 (Biolegend, clone G10F5). Live dead staining (ThermoFisher, Waltham, MA, USA) was performed as a control for vitality. Labelled cells were fixed in PBS−/− 1× 1% formalin. A minimum of 50.000 events were acquired for each sample using a BD Lyric (BD Biosciences) and analyzed by FACS Diva 8.0.1 (BD Biosciences).

### 2.4. Direct Infection of MDM and MN 

Differentiated MDM macrophages grown onto glass slides in 24-well tissue culture plates were infected with the three SARS-CoV-2 strains at 0.05 MOI/100 µL for 2 h at 33 °C, 5% CO_2_. After removal of the virus inoculum, cells were washed and maintained in serum-free Earl’s Minimal Essential Medium (EMEM) with the addition of 1% penicillin, streptomycin, glutamine and 5 γ/mL of trypsin. After 1, 4, 8, 24, 72 and 96 h, MDM were fixed in methanol/acetone 2:1 for 5 min and stained with the SARS-CoV-2 (2019-Cov) Nucleoprotein/NP antibody, Rabbit Mab (Sino Biological, Beijing, China) and anti-Spike antibody, Rabbit Mab (Sino Biological), followed by a donkey anti-rabbit Alexa Fluor 488 (ThermoFisher, Waltham, MA, USA). Nuclei were stained with DAPI (Sigma-Aldrich, St. Louis, MO, USA), and slides were examined at 40× with Nikon’s C1 Digital Eclipse Modular Confocal Microscope System (software Ez-C1, vers 3.70 Nikon Instruments Inc. Melville, NY, USA) for the expression of the N and S protein.

In parallel, supernatants obtained at the same time points were titrated in VERO E6 cells to detect complete virus replication with the production of the infectious virus. As a control, VERO E6 were infected in parallel with the same MOI of the three viral strains, and supernatants were titrated. The same experiments were performed also with MN.

### 2.5. MDM and MN Mediated Transmission of the Infectious Virus through Co-Culture with Infected VERO E6

**Figure 1:**
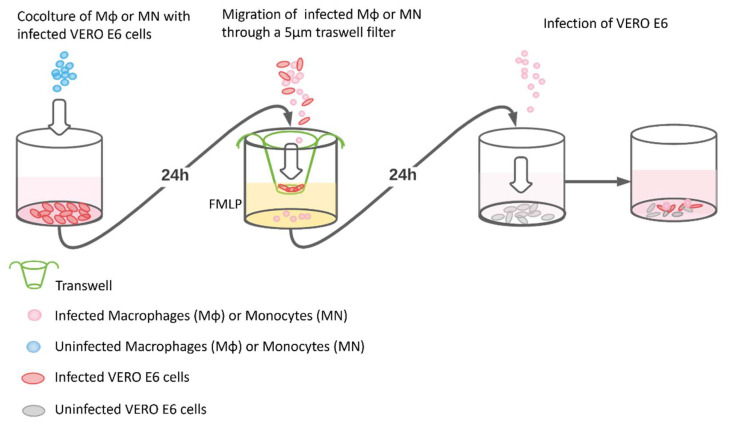
A schematic representation of experimental design used in the study. In the first well, MDM or MN were co-cultivated with infected VERO E6. In the second well, the co-cultured MDM/MN-VERO E6 cells were gathered and seeded on a 5 µm-filtered membrane (transwell) in the presence of the chemoattractant FMLP. Only the infected MDM/MN are able to migrate through the transwell, while the VERO E6 remained on the filter. Then, in the third well, the infected and migrated MDM/MN were transferred onto a new VERO E6 monolayer where they were able to transmit the infection (fourth well).

MDM and MN were co-cultured with VERO E6 cells previously infected with the three viral strains (Figure 1). The unequivocal demonstration that MDM and MN co-cultured with SARS-CoV-2 infected cells did not only express N and S antigens, but could also transmit the infectious virus, required the removal of all SARS-CoV-2 infected VERO E6 cells present in the co-culture. This was achieved by taking advantage of the capacity of MDM and MN to migrate through natural and artificial barriers in response to chemotactic agents such as the N-formylmethionyl-leucyl-phenylalanine (FMLP) peptide [12]. Control experiments showed that VERO E6 infected or mock-infected were not able to migrate in response to the FMLP and pass through the 5 µm pore. The experiment was conducted as follows: 3 × 10^5^ VERO E6 cells were seeded in 24-well microplates for 72 h and infected with the three strains of SARS-CoV-2 at 0.05 MOI for 1 h at 33 °C, 5% CO_2_. The inoculum was removed, and 1 mL of serum-free EMEM with the addition of 1% penicillin, streptomycin, glutamine and 5 γ/mL of trypsin were added. Then, 48 h post infection, 5 × 10^6^ MDM and MN were separately added to each well and co-cultured overnight. The day after, cells were collected, washed and allowed to migrate for 3 h through a 5 µm transwell filter (Costar—Corning Incorporated, NY 14831, USA), which allows the passage of only MDM and MN in response to the 10^−8^ FMLP chemoattractant. Migrated cells were washed with trypsin EDTA to detach the virus potentially attached to the cell surface and were abundantly washed with PBS, and 2 × 10^5^ MDM and MN were used to prepare cytospin slides [15]. The same amount of migrated cells was also inoculated into permissive VERO E6 cells for virus isolation and observed every other day for the development of the cytopathic effect (CPE). Supernatants from the last wash were inoculated onto VERO E6 cells as a control to verify the complete virus removal.

### 2.6. Virus Transmission Assay

To demonstrate that virus transmission from infected MDM to VERO E6 cells was mediated by cell membrane fusion with cytoplasmic material exchange, two vital fluorescent dyes were used: 5-chloromethylfluorescein diacetate (CMFDA, Invitrogen, Waltham, MA, USA) and 4-({[4-(chloromethyl)phenyl]carbonyl}amino)-2-(1,2,2,4,8,10,10,11-octamethyl-10,11-dihydro-2H-pyrano[3,2-g:5,6-g’]diquinolin-1-ium-6-yl)benzoate (CMTPX, Invitrogen). Each dye was used as a 5 mM stock solution in dimethylsulfoxide (Merck, Kenilworth, NJ, USA). Overall, 2 × 10^5^ infected and uninfected MDM were stained with 1 mM CMTPX (Cell Tracker Red), whereas 3 × 10^5^ VERO E6 were stained with 1 mM CMFDA (Cell Tracker Green) according to a reported procedure [16] and seeded in a 24-well microplate for 24 h. The day after, infected or uninfected red MDM were inoculated onto uninfected green VERO E6 and incubated for 24 h at 33 °C, 5% CO_2_. The microscopic observation of VERO E6 cells and their dual fluorescing fusion products (orange/yellow merging color) was achieved by using a fluorescent microscope (model DM RBE; Leica, Wetzlar, Germany) with filter G/R DM 513803 (Leitz) designed for simultaneous excitation by 490 +/− 20 and 575 +/− 30 nm fluorescent light.

### 2.7. Kinetic of N and S Protein Expression and RNA Replication on MDM, MN and VERO E6

The kinetic of N and S expression in MDM was evaluated with a confocal microscope after 1, 4, 8, 24, 48, 72 and 96 h, whereas MN were observed only with a fluorescent microscope (model DM RBE; Leica, Wetzlar, Germany). In parallel, infected MDM and MN at the same time-course were detached, washed and frozen for RNA quantification in real-time RT-PCR targeting the E gene according to WHO guidelines [17]. Moreover, VERO E6 cells were infected with the same amount of the virus (0.05 MOI) and trypsinized, washed and frozen to be tested at the same time points. To compare the amount of RNA replication in MDM, MN and VERO E6, the ß2 globulin was quantified to calculate the exact number of cells tested. SARS-CoV-2 RNA was quantified by real-time RT-PCR in supernatants from each time point from MDM, MN and VERO E6 cells. 

### 2.8. Mechanism of Virus Entry into MDM and MN

To investigate whether only the mechanism of receptor binding was involved in the virus entry into MDM and MN, the virus was treated with a pool of human post-COVID-19 sera with a SARS-CoV-2 neutralizing titer higher than 1:640 and with a bi-specific human monoclonal antibody anti-S protein [18]. MDM and MN were differentiated as previously reported in 24-well microplates. In addition, VERO E6 cells were seeded for the control. One hour before the experiment, 100 µL of the virus preparation at 0.05 MOI were mixed with 100 µL of the post-COVID-19 serum pool at a dilution of 1:10 to 1:640 and with 0.7 µg/mL of a bi-specific human anti-spike monoclonal antibody and incubated at 33 °C, 5% CO_2_. These mixtures were then added to MDM, MN and VeroE6 cells for 1 h at 33 °C; then, the inocula were removed, and EMEM was added to each well. After 24 h, the cells were stained for the expression of the N protein to verify virus entry. The untreated virus was used as a control for MDM, MN, and VERO E6.

### 2.9. Light Microscopy and Light Microscopy Immunohistochemistry

For light microscopy, cell pellets were fixed in a 10% buffered formalin solution, dehydrated and embedded in paraffin. Serial sections, 3 mm thick, were cut and stained with hematoxylin-eosin; unstained sections were used for immunohistochemistry. Sections were de-paraffinized and brought to a TRIS phosphate-buffered saline solution (TBS O, l5 M, pH 7.35). After blocking the endogenous peroxidase with 3% H_2_O_2_, and pretreatment with trypsin 0.05%, the slides were incubated overnight with anti-spike and anti-nucleocapsid antibodies (Table 1). The immunohistochemical staining was performed with the peroxidase-antiperoxidase method, and diaminobenzidine was used as the chromogen substrate. Specificity tests were performed using appropriate negative controls.

### 2.10. Ultrastructural Study

For electron microscopy, samples were fixed with Karnovsky’s solution in a cacodylate buffer 0.2 M (pH 7.3) for 4 h at 4 °C, then postfixed with 1% osmium tetroxide in the cacodylate buffer 0.2 M (pH 7.3) for 1 h at RT, dehydrated in ethanol and propylene oxide and embedded in epon-araldite resin. Ultrathin sections were stained with uranyl acetate and Reynolds’s lead citrate and observed with a JEOL JEM 1011 electron microscope. Pre-embedding immunogold staining was performed in infected VERO E6 cells at 72 h from inoculation to validate the specificity of the commercial antibodies. The pellet containing VERO E6 cells in PBS solution was subjected to three washing cycles in Tris buffer (10 min each), immersed in 0.05% TritonX for 20 min, washed in Tris buffer, threatened in 10% Normal Goat Serum (30 min) and incubated with the primary antibody (1:50 for 92 h at 4 °C). After five washing cycles in Tris buffer (10 min each), the pellet was incubated with the secondary antibody conjugated to 15 nm gold particles (72 h, 4 °C) (BBI Solutions), washed in Tris buffer, fixed in Karnovsky’s solution (15 min, 4 °C), post-fixed in 1% osmium tetroxide (1 h, room temperature), washed in Tris buffer and dehydrated in a graded series of ethyl alcohols. Finally, the specimen was embedded in Epon-Araldite overnight at 60 °C. Ultra-thin sections were stained with uranyl acetate 5% and Reynold’s solution, and observed using a JEOL JEM 1011 electron microscope.

### 2.11. Statistical Analysis

Descriptive data were reported or considered as absolute and relative frequencies or the mean and confidence interval 95% (CI 95%) based on the type of the variable distribution. Analyses were performed using the GraphPad Prism 8.3.0 (GraphPad Software, La Jolla, CA, USA).

## 3. Results

### 3.1. Analysis of Macrophages and Monocytes Infected with Different SARS-CoV-2 Variants

**Figure 2:**
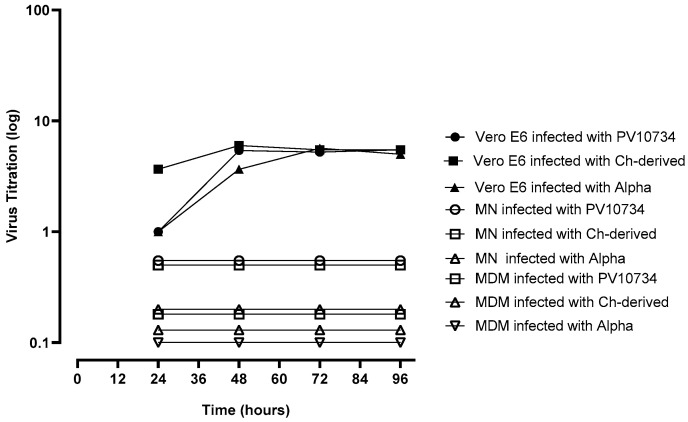
Time course of virus production in supernatants from monocytes-derived macrophages (MDM), monocytes (MN) and VERO E6 infected with three virus variants. The experiments were performed in triplicate.

After the incubation of cell-free virus preparations with MDM and MN, the N protein was detected in both cell types with all the three SARS-CoV-2 strains. However, no CPE was observed after the inoculation of supernatants onto VERO E6 cells at each time point of the experiment (since 24 to 96 h post infection). On the other hand, supernatants of the three viral strains inoculated onto VERO E6 cells and collected at the same time points gave CPE after the subsequent inoculation onto new VERO E6 cells; the growth curves of the virus titer are shown in Figure 2.

**Figure 3:**
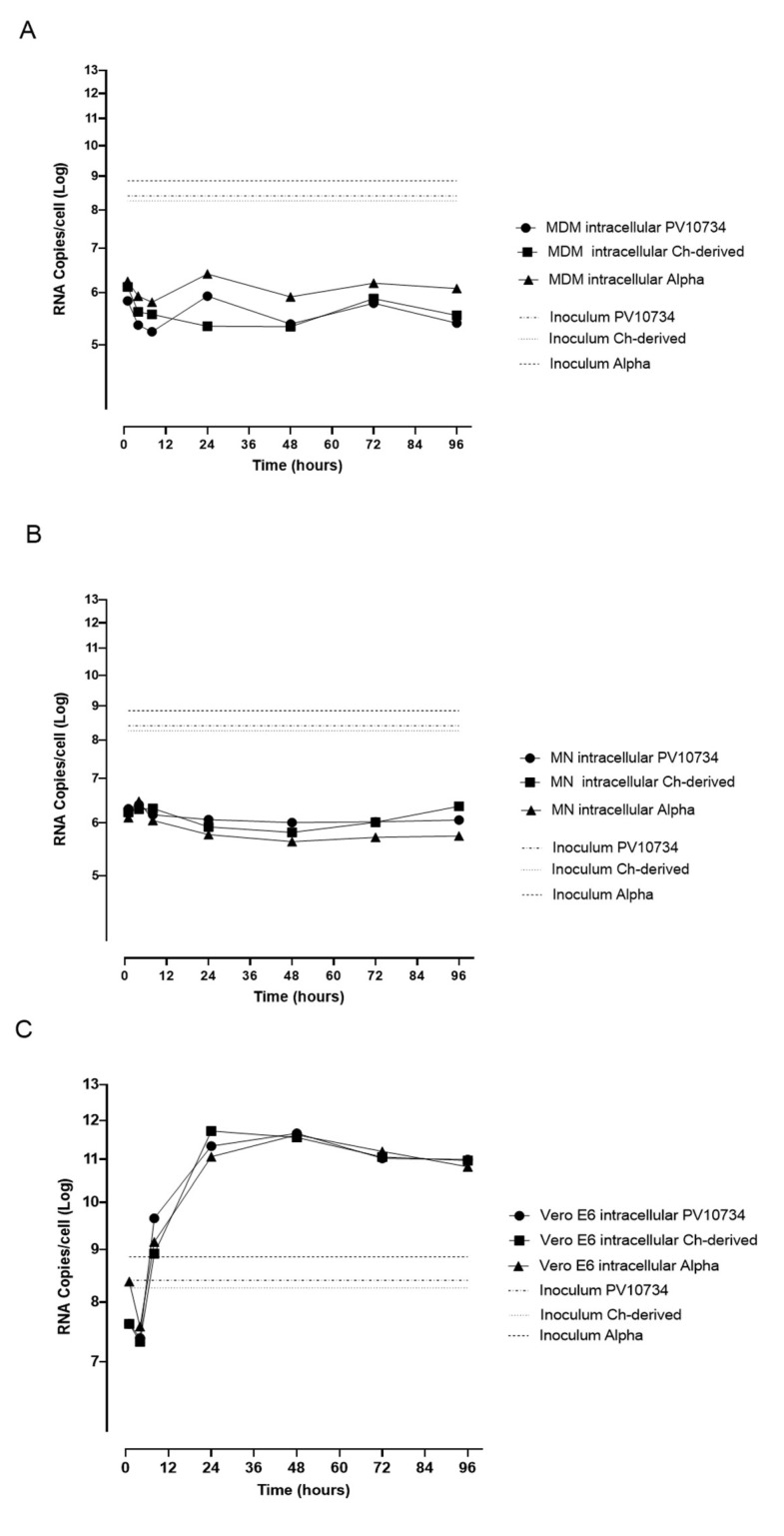
Time-course for RNA quantification in infected MDM (**A**), MN (**B**) and VERO E6 (**C**) with the three virus strains at different time points. Virus input used for infection is reported. The experiments were performed in triplicate.

To investigate the absence of viral replication in MDM and MN further, we quantified the intracellular RNA in these cells after infection with the three variants (Figure 3A,B) in comparison to that produced in VERO E6 (Figure 3C). MDM intracellular SARS-CoV-2 RNA was negligible and did not change at each time point (median copy number 2.4 × 10^5^ Italian strain, 3.7 × 10^5^ Chinese strain and 1.2 × 10^6^ Alpha variant; *p* = 0.0012), indicating a lack of viral synthesis. In contrast, intracellular SARS-CoV-2 RNA in VERO E6 increased during the time point (median copy number 9.9 × 10^10^ Italian strain, 9.4 × 10^10^ Chinese strain and 6.6 × 10^10^ Alpha variant). Intracellular median SARS-CoV-2 RNA in MDM was 5 log lower than in VERO E6 (*p* < 0.0001), confirming that no complete viral replication was present in MDM even if a median of 10^6^ copies of viral RNA was maintained, while infection was productive only in VERO E6. Similar results were obtained for MN.

### 3.2. Analysis of MDM and MN Infection Co-Cultured with Infected VERO E6

**Figure 4:**
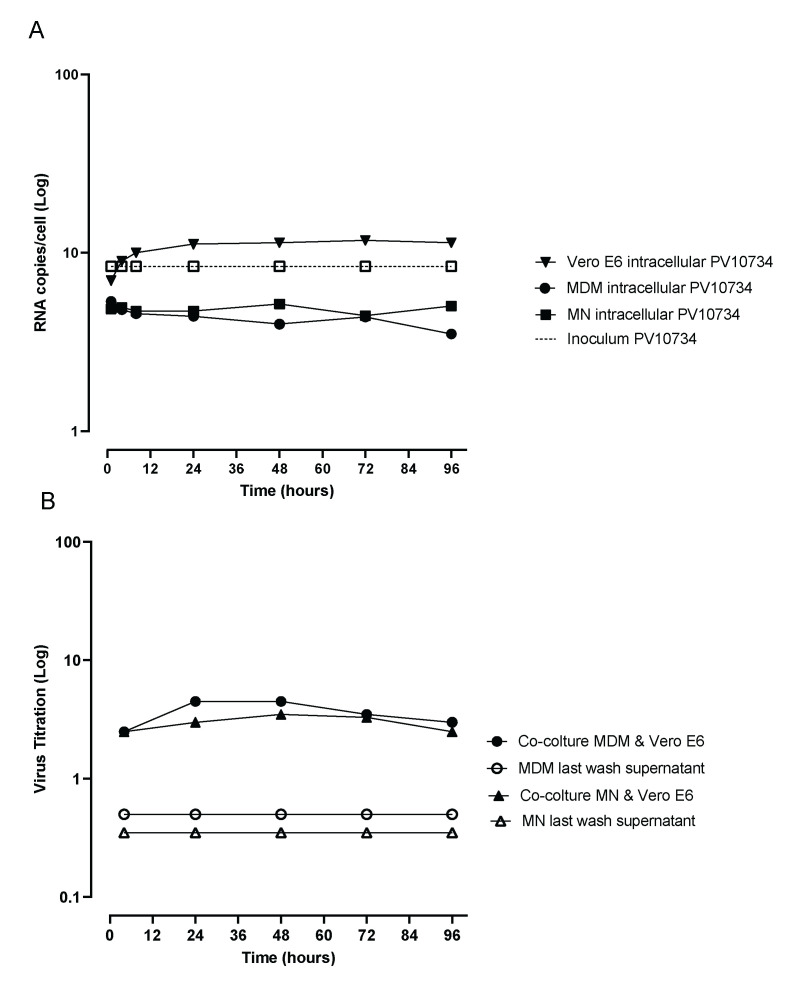
Time-course of intracellular RNA quantification in MDM and MN in comparison with VERO E6 (**A**) and virus release in supernatants (**B**) from MDM and MN infected with Italian strain and co-cultivated with VERO E6 at different time points in comparison with supernatants of the last wash of the same cells inoculated into VERO E6. The experiments were performed in triplicate.

MDM and MN, co-cultured overnight with VERO E6 infected with the Italian SARS-CoV-2 strain, were cleared from infected VERO E6 by means of migration through a 5 µm filter. The migrated cells internalized the virus during contact with the infected VERO E6 and were able to transmit the virus when inoculated onto naïve VERO E6, mimicking a phenomenon that can occur in vivo in infected patients. CPE was detected as soon as 48 h post-infection (p.i.) on VERO E6 and increased until the complete detachment of all the cells at 96 h p.i. The titration of virus production in the supernatant and intracellular RNA quantification are reported in Figure 4.

Supernatants from the last wash of the co-cultured MDM and MN inoculated as controls in VERO E6 cells did not show CPE, confirming the complete removal of the virus not internalized into the cells. Virus transmission from migrated MDM and MN to VERO E6 was detected at all time points. When we looked at the mechanism of virus entry into MDM and MN, we found that the virus treated with the pool of post-COVID 19 patients’ sera and the anti-Spike monoclonal antibody was detected into MDM and MN stained for the N protein in an equal number of cells as in the untreated control, showing that also the other mechanism that did not involve the binding of Spike to its receptor is probably involved in virus entry.

### 3.3. Virus-Induced Fluorescent Probe Transmission Assay

**Figure 5:**
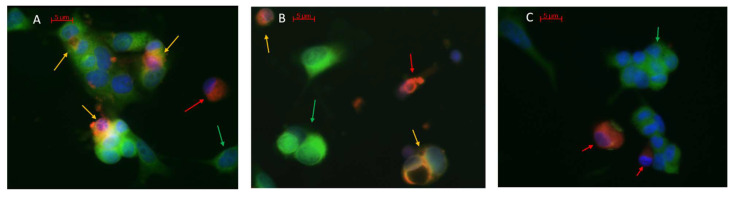
Virus transmission assay of: (**A**) infected MDM, (**B**) infected MN stained in red (red arrow) co-cultured with uninfected VERO E 6 stained in green (green arrow). The merging orange color (yellow arrows) is representative of virus transmission to VERO E6. In blue, the nuclei stained with DAPI. (**C**) Uninfected MDM stained in red (red arrow) co-cultured with uninfected VERO E6 stained in green (green arrow).

Infected MN and MDM labelled with a red fluorescent probe transmitted the dye to uninfected VERO E6 labelled with the green probe, resulting in the fusion of the two fluorocromes with a merging orange/yellow color. This fusion was observed starting from 24 h after the co-cultivation of infected MN and MDM with uninfected VERO E6 (Figure 5A,B). No fusion of fluorescent probes was observed in VERO E6 co-cultured with uninfected MDM (Figure 5C).

### 3.4. Kinetics of N Protein Uptake by MDM and MN after Co-Cultivation

MDM and MN infected during co-cultivation with VERO E6 infected with the three viral strains were purified by migration through the transwell filter. Subsequently, MDM and MN were examined for N and S protein expression after 1, 8, 24, 48, 72 and 96 h. N antigen expression was detected as early as 1 h in 30% of MDM. The number of positive cells increased, involving all the cells present in the slides 8 h p.i., with an increase also in the intensity of the staining for the N protein. MDM start to become larger, and some oval structures appeared in the nuclei, in parallel with some other structures located outside of MDM that we called “blackberry-shaped” structures. These structures appeared as soon as 8 h p.i for the Chinese strain and from 24 h p.i. for the other two strains. These structures increased in number in parallel with the enlargement of MDM as reported in Table 2.

**Figure 6:**
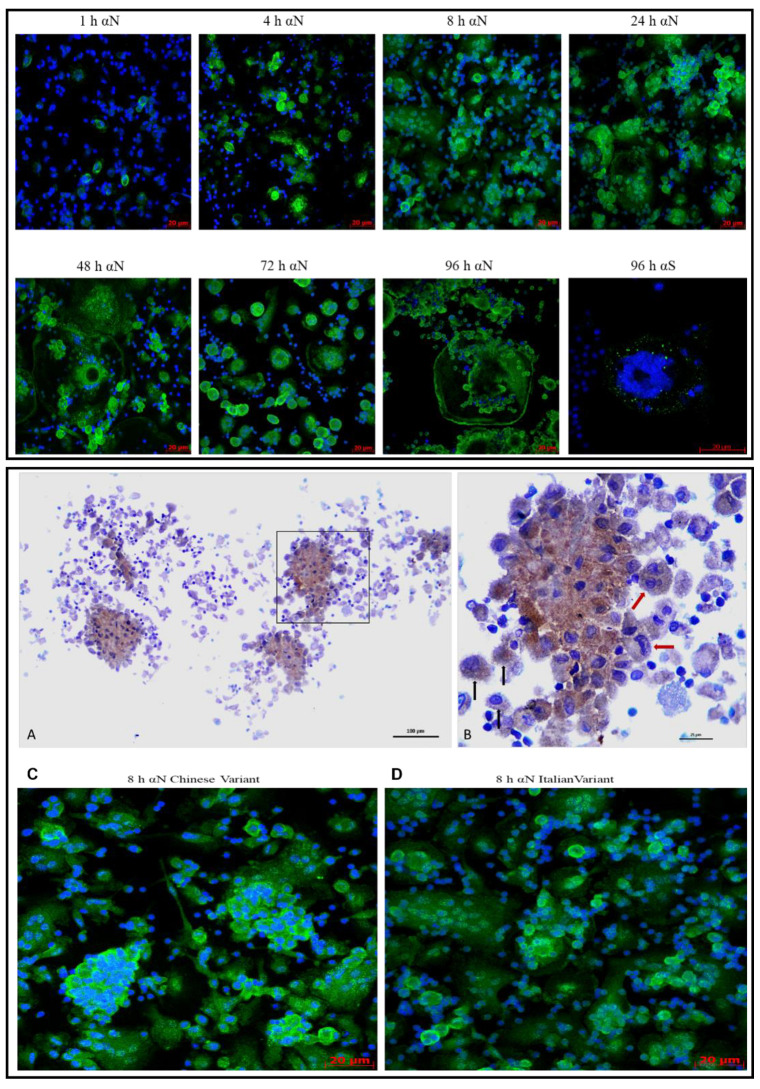
Time-course of MDM infected with the SARS-CoV-2 strains/variant. The upper panel shows the Italian strain time-course. Cells are stained with the anti-N and anti-Spike protein (green fluorescence) and with DAPI (blue fluorescence), at 40× magnification in confocal microscopy. The lower panel shows the anti-N immunostaining (brown stain) of infected MDM (**A**,**B**) and the comparison of “blackberry-shaped” formations in the Chinese-derived (**C**) and PV10734 strains (**D**) 8 h p.i. with confocal microscopy. The area squared in (**A**) is enlarged in panel (**B**) with multinucleated MDM (red arrows) and individual cells (black arrows).

From 72 to 96 h p.i., the number of MDM decreased because the engulfed cells lysed with an increase of “blackberry-shaped” structures. The survived MDM increased their dimension by 20–40 times in comparison with 1 h p.i. Spike protein expression was detected only 96 h p.i. The above characteristics and the time-course of these events in MDM are reported in Table 2 and Figure 6. In panel A and B, the anti-nucleocapsid immunostaining of infected MDM fixed in formalin and embedded in paraffin demonstrates that this immunostaining can be used on routinely processed samples for pathology. Immunostaining specifically labels the cytoplasm, and shows a punctated morphology, with single cells that tend to coalesce, appearing as multinucleated (red arrows, B). The MDM activation is morphologically supported by the large number of pseudopodia that confer a hairy-like appearance to non-coalescent individual cells (black arrows, B).

**Figure 7:**
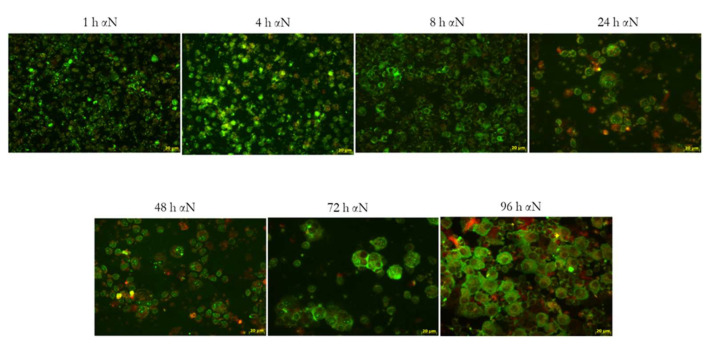
Time-course of MN infected with the SARS-CoV-2 Italian strain. In the panel is reported the PV10734 strain time course stained with the anti-N (green fluorescence) at 40× magnification in fluorescent microscope.

Similar results were obtained with MN (Figure 7): 1 h p.i., a lower number of cells (10%) were infected compared to MDM and increased in the number and intensity of the N protein expression. Eight h p.i., all the cells were infected and at 72/96 h became bigger. No “blackberry-shaped” forms were detected at any time point examined. No differences were detected in the morphology of the MN infected with the three variants.

### 3.5. Pathologic Study of Infected MDM and VERO E6 Cells

**Figure 8:**
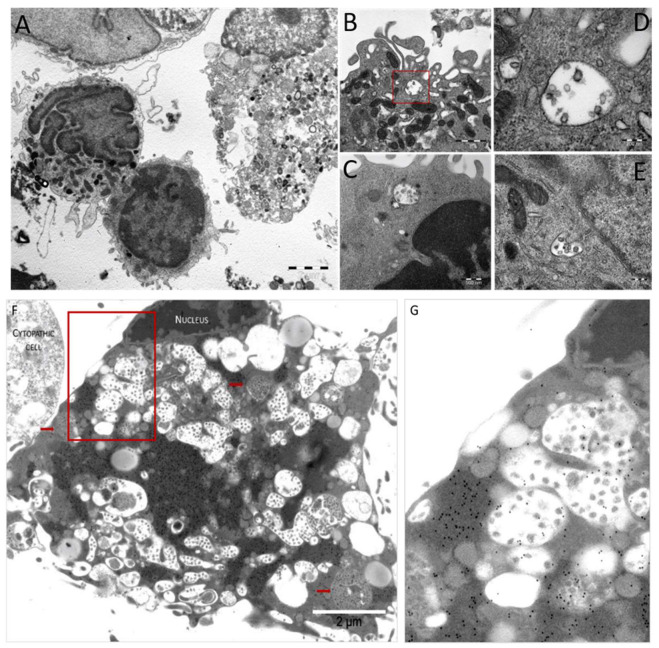
Electron micrograph showing features that recapitulate all morphofunctional characteristics of in vitro SARS-CoV-2 infected macrophages (**A**–**E**) and VERO E6 (**F**). (**A**) Low magnification view of in vitro infected macrophages at 24 h p.i. (**B**) Red squared area—intracytoplasmic vesicles containing viral particles, enlarged in panel (**C**–**E**). Examples of vesicles in macrophages that still preserve their morphologic integrity but show features of cell activation (pseudopodia) (**D**) and preserved organelle morphology (**E**,**F**). Pre-embedding anti-nucleocapsid immunostaining of VERO E6 cells at 72 h p.i. with multiple replication vesicles and loose viral particles (those within the vesicles with the white background) or more compacted vesicles (red arrows) with multiple smaller virions. The red squared area in panel F is enlarged in panel **G**, highlighting the gold-particle-free membrane of the macrophage.

The light microscopy study conducted at the different intervals from the viral inoculation on MDM showed early syncytial-like cells and later (>24 h) the aggregation of multiple cells, resulting in the “blackberry-shaped” structures observed with fluorescence studies, as shown in the upper panel of Figure 6. The ultrastructural study of infected MDM demonstrated: (i) intracytoplasmic vesicles containing viral particles (persistence vesicles). These MDM showed the activation and preservation of cytoplasmic membranes; (ii) the absence of replication vesicles similar to those observed in infected VERO E6 cells (Figure 8F). The intracytoplasmic vesicles, each containing a few viral particles, were observed in MDM infected after 1 h, as well as after 24, 48, 72 and 96 h from virus inoculation, suggesting that viral particles included in membrane-bounded vesicles persist, irrespective of the interval from viral inoculation in MDM (Figure 8A–E). Indeed, in infected VERO E6 cells, viral particles were Too Many To Count (TMTC), both free in the cytoplasm and clustered in replication vesicles. In addition, infected VERO E6 cells showed numerous viral particles adherent to cell membranes, with a high concentration on cytoplasmic pseudopodia (Figure 8F). The pre-embedding immunoelectron microscopy study shows specific anti-nucleocapsid immunostaining of both viral particles and nucleocapsid material free in the cytoplasm, with a high amount of viral proteins and a low number of mature viral particles (Figure 8F). The dark osmiophilic cytoplasmic protein masses that are specifically labelled by the anti-nucleocapsid antibodies demonstrate that actively infected VERO E6 cells produce more viral proteins than viral particles. The small light gray part of a cytopathic adjacent cell (Figure 8F upper left corner) also shows protein immunolabeling without the detection of particles. The specificity of the immunostaining is proven by the absence of immunogold particles in the extracellular background (Figure 8F). This feature was not observed in infected MDM (Figure 8A–E).

## 4. Discussion

Our study unravels the bidirectional trajectory of reciprocal infection between VERO E6 cells and MDM/MN. In this trajectory, infected VERO E6 cells with active viral replication transmit the virus to both MDM and MN, where the virus persists but does not replicate. In the opposite direction, infected MDM and MN with a persistent but not replicating virus infect VERO E6 cells that demonstrate active viral replication. Therefore, MDM and MN can host the viable virus but are not permissive for viral replication. These in vitro observations confirm that MDM could be key players of viral dissemination and persistence in circulating MN cells that can transfer viable viruses when co-localizing with epithelial cells.

Several authors showed that SARS-CoV-2 can infect MDM and MN without virus production [11], but the transmission of the virus from infected MDM is still debated. To the best of our knowledge, this is the first study reporting that MDM and MN infected by different variants of SARS-CoV-2 can transmit the infection to permissive VERO E6 cells acting as a “Trojan horse” in in vivo infection, spreading out the virus to target cells in different body sites. In the presence of all components of viral binding and activation, the virus can infect MDM and MN without replication, stimulating the production of proinflammatory cytokines and chemokinesas described in Mers-CoV [10]. Upon infection, MN migrate into tissue and become infected resident MDM, enabling the virus to infect susceptible cells. Infected MDM, paradoxically, facilitates the invasion of different organs by SARS-CoV-2, probably inducing in parallel with protracted local and systemic inflammatory responses in multiorgan failure.

We demonstrated that MDM and MN are abortively infected by SARS-CoV-2 both directly or through co-cultivation with infected VERO E6. We already showed this mechanism in HCMV infection. In that case, PMNL could be infected only through co-cultivation with infected endothelial primary cells without the replication and production of the virus by PMNL, whereas they were able to transmit the infectious virus to uninfected endothelial cells [12]. These observations indicate that virus infectivity can be preserved inside MDM and MN as in PMNL for HCMV, even if these cells are not permissive for virus replication. Indeed, we detected by EM a discrepancy between the amount of viral proteins (high) and mature viral particles (low), suggesting that the virus prompts the infected cell to produce more viral proteins than mature viral particles, which are, however, sufficient to transmit the infection to the target cells. The increase in viral proteins may be a consequence of an initial but abortive replication. Moreover, in agreement with ex vivo observations in thrombi, co-cultured MDM were shown to carry viral particles, and we could also detect viral RNA that did not increase during the time-course, confirming our hypothesis of viral persistence but a lack of productive replication inside these cells.

The kinetics of the expression of the N protein in the time-course showed that just after 8 h, all the MDM were infected by the three variants with increasing fluorescence and the fragmentation of the nuclei and formation of ”blackberry-shaped” structures full of viral proteins. These ”blackberry-shaped” formations start to appear as early as 8 h with the Chinese strain and in the next 24 h for the other variants. We can speculate that these structures could be identified as microvesicles in coalescent cells that are produced and released at a high concentration in inflammatory conditions [19]. These microvesicles, at the confocal microscopy, react with the SARS-CoV-2 N protein and could act as extracellular shuttles for the virus dissemination. The kinetics of N protein expression in the MN time-course showed a lower amount in the cells’ number of the infection after 1 h compared to MDM, but no differences were detected 8 h p.i. The main morphological difference was the lack of blackberry-shaped forms, even if also in MN an enlargement of cells was observed between 24 and 48 h p.i. Moreover, MN were infected with the three viral variants and did not show any morphological differences.

Looking at viral transmission to VERO E6 from infected MDM and MN through co-cultivation, or direct infection in the time-course, we could isolate the three variants at each time point. The electron microscopy staining with gold particles confirms the absence of viral particles adhering to the cell membrane of infected cells, excluding a contamination of the cell surface.

These data show that the virus survives inside MDM and MN and, even if it does not replicate, it spreads in different body districts. The viral load detected in the time-course in MDM/MN and VERO E6 confirms our hypothesis that the virus actively replicates in permissive VERO E6, but does not replicate in MDM and MN.

ACE2 is expressed in multiple cell populations that can be found in the lungs, including alveolar type II pneumocytes and macrophages [19,20], suggesting that SARS-CoV-2 can potentially infect ACE2+ macrophages. HCoV-229E can infect MDM as they express the aminopeptidase N(APN) receptor needed for viral entry and bypass the endosome to enter the target cell by using TMPRSS2 [20,21,22]. The endosomal pathway in MDM is critical to identify invading pathogens, allowing HCoV-229E to infect MDM without triggering an antiviral response, thus enhancing its pathogenesis. MDM infected with HCoV-229E undergo cell death due to the lytic release of new viral particles [20], suggesting that HCoV-229E can infect and replicate in MDM. Similar to HCoV-229E, SARS-CoV-2 can also infect MDM by phagocytosis and can be detected in phagolysosomes of infected human macrophages [23,24].

SARS-CoV-2 infection of its target cells depends on ACE2 and TMPRSS2 receptors [25], similarly to HCoV-229E, which uses TMPRSS2 receptor for cell entry.

In our experiments, looking at the virus entry mechanism, we found that virus entry into VERO E6 was blocked by human neutralizing antibodies and anti-spike monoclonal antibodies that block the viral spike protein, avoiding the binding with the VERO E6 receptors. When we looked at MDM and MN, we found that the expression of the N protein was present into these cells even if the virus was blocked by the neutralizing antibody, showing that more than one mechanism is involved in the infection of these cells. This is in agreement with the recent study of Florian et al. [26] that demonstrated the involvement of the sialic acid-binding Ig-like lectin 1 (SIGLEC1) in enhancing ACE2-mediated infection, acting as attachment receptors rather than entry receptors for SARS-CoV-2. In conclusion, MN and MDM are pivotal innate immune cells with a crucial role in defensive activities against various antimicrobial agents such as viruses. Besides this beneficial activity, they can exert unfavorable effects, extending viral infection within the body. However, their role has not yet been definitely established. Here, we showed that SARS-CoV-2 infects MN and MDM without production of the infectious virus but preserving infectivity. Therefore, these cells may act as spreaders for the virus in different body districts as a “Trojan horse”.

## Figures and Tables

**Table 1:** Antibodies used through the experiments.

Antibody	Dilution	Pretreatment	Secondary	Code	Specificity
SARS-CoV-2 Spike S1	1:200	2 cycles MW (900 W) + 3 cycles MW (720 W), pH 9.9	DR	Sino Biological 40150-R007	2019-nCoV S
SARS-CoV-2 Nucleocapsid	1:2500	2 cycles MW (900 W) +3 cycles MW (720 W), pH 9.9	DR	Sino Biological 40143-R019	2019-nCoV N
CD68 (PGM1)	1:100	15’ Trypsin	DM	Dako M0876	Subcellular fraction of human MØ
CD68 (KP1)	1:100	15’ Trypsin	DM	Santa Cruz SC-20060	Subcellular fraction of human MØ
CD163	1:200	no	DM	ThermoFisher Scientific MA5-11458	Human MN and MØ. Circulating MN and most tissue MØ

Legend: DR: Donkey-rabbit; DM: Donkey-Mouse; 2019-nCoV: novel coronavirus 2019; S: Spike; N: Nucleoprotein; MN: monocytes; MØ: macrophages.

**Table 2:** Characteristics of infected MDM and MN in I detected during the time-course.

MDM															
Characteristics	Virus Variant/ Lineage	1 h		4 h		8 h		24 h		48 h		72 h		96 h	
		Mean	95% CI	Mean	95% CI	Mean	95% CI	Mean	95% CI	Mean	95% CI	Mean	95% CI	Mean	95% CI
Infected Cells	B.1.1/Alpha	30%	19.1–41.5	72%	55.1–88.2	TMTC	na	TMTC	na	TMTC	na	TMTC	na	TMTC	na
	Chinese	33%	26.2–40.5	81%	71.6–90.7	TMTC	na	TMTC	na	TMTC	na	TMTC	na	TMTC	na
CPE	B.1.1/Alpha	-	na	-	na	-	na	±	na	+	na	+	na	+	na
	Chinese	-	na	-	na	±	na	±	na	+	na	+	na	+	na
Average Cell dimension (µm^2^)	B.1.1/Alpha	562.6	441.0–684.2	837.5	620–1056	1262	1029–1495	2246	1317–3175	4521	2640–6403	6009	3774–8544	11522	3197–26240
	Chinese	189.3	131.8–246.7	628.2	550.3–706.2	906.9	653–1161	2416	1742–3090	3750	2279–5222	5004	2900–7107	8509	3095–13923
«blackberry-shaped» forms *	B.1.1/Alpha	0	na	0	na	0	na	1	0.76–2.1	2	0.9–3.8	2	0.3–3	2	0.9–3.8
	Chinese	0	na	0	na	2	0.9–3.8	5	0.03–9	2	0.9–3.8	2	0.4–4.5	2	0.9–3.8
Multinucleated Cells	B.1.1/Alpha	<10%	na	<10%	na	<10%	na	32%	26.5–36.8	48%	34.2–62.5	51%	33.2–68.1	51%	33.2–68.1
	Chinese	<10%	na	12%	8.8–14.5	33%	22.2–43.8	29%	19.7–38.1	54%	14.4–92.0	50%	36.0–63.4	69%	50.0–87.3
Spike protein	B.1.1/Alpha	-	na	-	na	-	na	-	na	-	na	-	na	+	na
	Chinese	-	na	-	na	-	na	-	na	-	na	-	na	+	na
MN															
Infected Cells	B.1.1/Alpha/Chinese	11%	4.299–17.06	75%	64.39–86.55	TMTC	na	TMTC	na	TMTC	na	TMTC	na	TMTC	na
CPE	B.1.1/Alpha/Chinese	-	na	-	na	-	na	±	na	+	na	+	na	+	na
Average Cell dimension (µm^2^)	B.1.1/Alpha/Chinese	31.92	25.01–38.83	50.98	45.33–56.63	57.9	48.74–67.06	117.7	72.28–163.2	133.4	110.0–156.8	259	186.9–331.1	215.4	148.5–282.3
«blackberry-shaped» forms *	B.1.1/Alpha/Chinese	0	na	0	na	0	na	0	na	0	na	0	na	0	na
Multinucleated Cells	B.1.1/Alpha/Chinese	<10%	na	<10%	na	<10%	na	<10%	na	0	na	4.00%	1.221–6.001	11%	4.263–16.88
Spike protein	B.1.1/Alpha/Chinese	-	na	-	na	-	na	-	na	-	na	-	na	+	na

* «blackberry-shaped» formations per field; TMTC: Too Many To Count; CPE: cytopathic effect; 95% CI: 95% confidence interval.

## Data Availability

The data presented in this study are available on request from the corresponding author.
